# Associations between a range of enteric methane emission traits and performance traits in indoor-fed growing cattle

**DOI:** 10.1093/jas/skae346

**Published:** 2024-11-08

**Authors:** Sean B Crowley, Deirdre C Purfield, Stephen B Conroy, David N Kelly, Ross D Evans, Clodagh V Ryan, Donagh P Berry

**Affiliations:** Department of Animal Bioscience, Animal and Grassland Research and Innovation Centre, Teagasc, Moorepark, Fermoy, County Cork, Ireland; Department of Biological Sciences, Munster Technological University, Bishopstown, County Cork, Ireland; Department of Biological Sciences, Munster Technological University, Bishopstown, County Cork, Ireland; Irish Cattle Breeding Federation, Link Road, Ballincollig, County Cork, Ireland; Irish Cattle Breeding Federation, Link Road, Ballincollig, County Cork, Ireland; Irish Cattle Breeding Federation, Link Road, Ballincollig, County Cork, Ireland; Irish Cattle Breeding Federation, Link Road, Ballincollig, County Cork, Ireland; Department of Animal Bioscience, Animal and Grassland Research and Innovation Centre, Teagasc, Moorepark, Fermoy, County Cork, Ireland; Department of Animal Bioscience, Animal and Grassland Research and Innovation Centre, Teagasc, Moorepark, Fermoy, County Cork, Ireland

**Keywords:** beef cattle, Greenfeed, residual methane, sustainable agriculture, greenhouse gas

## Abstract

Despite the multiple definitions currently used to express enteric methane emissions from ruminants, no consensus has been reached on the most appropriate definition. The objective of the present study was to explore alternative trait definitions reflecting animal-level differences in enteric methane emissions in growing cattle. It is likely that no single methane trait definition will be best suited to all intended use cases, but at least knowing the relationships between the different traits may help inform the selection process. The research aimed to understand the complex inter-relationships between traditional and novel methane traits and their association with performance traits across multiple breeds and sexes of cattle; also of interest was the extent of variability in daily enteric methane emissions independent of performance traits like feed intake, growth and liveweight. Methane and carbon dioxide data were collected using the Greenfeed system on 939 growing crossbred cattle from a commercial feedlot. Performance traits including feed intake, feeding behavior, liveweight, live animal ultrasound, subjectively scored skeletal and muscular traits, and slaughter data were also available. A total of 13 different methane traits were generated, including (average) daily methane production, 5 ratio traits and 7 residual methane (**RMP**) traits. The RMP traits were defined as methane production adjusted statistically for different combinations of the performance traits of energy intake, liveweight, average daily gain, and carcass weight; terms reflecting systematic effects were also included in the fixed effects linear models. Of the performance traits investigated, liveweight and energy intake individually explained more of the variability in methane production than growth rate or fat. All definitions of RMP were strongly phenotypically correlated with each other (>0.90) as well as with methane production itself (>0.86); the RMP traits were also moderately correlated with the methane ratio traits (>0.57). The dataset included heifers, steers, and bulls; bulls were either fed a total mixed ration or ad lib concentrates. When all sexes fed total mixed ration were compared, bulls, on average, emitted the most enteric methane per day of 269.53 g, while heifers and steers produced 237.54 and 253.26 g, respectively. Breed differences in the methane traits existed, with Limousins, on average, producing the least amount of methane of the breeds investigated. Herefords and Montbéliardes produced 124.50 g and 130.77 g more methane per day, respectively, than Limousins. The most efficient 10% of test-day records, as defined by daily methane independent of both energy intake and liveweight emitted, on average, 54.60 g/d less methane than animals that were average for daily methane independent of both energy intake and liveweight. This equates to 6.5 kg less methane production per animal over a 120-d finishing period for the same feed intake and liveweight.

## Teaser

This study explores established and novel enteric methane trait definitions in growing crossbred cattle, aiming to unravel associations between various methane traits and economically important performance traits, while also quantifying inter-breed differences.

## Introduction

A sustainable food system is defined as a system that preserves the economic, social, and environmental foundations needed to sustain food security and nutrition for generations to come (Story et al., 2009; Fao, 2014). Livestock production systems and, in particular the beef sector, have been subjected to intensifying economic, social and environmental pressures owing to growing costs (Hocquette et al., 2018) coupled with an intensifying interest among consumers concerning the environmental footprint of such systems (Rojas-Downing et al., 2017). Methane emissions are of primary interest to consumers as livestock accounts for one third of total anthropogenic methane emissions globally (Zhang et al., 2022). Several methane mitigation strategies are being explored but, for such mitigation strategies to be acceptable by producers, they must not only be cost-effective but also have no unfavorable repercussions on the sustainability of food systems. Of particular importance to the profitability of the beef cattle sector is animal growth and carcass value which have been documented to have an undesirable (positive) relationship with methane production (Lakamp et al., 2022).

Breeding programs to reduce enteric methane output of cattle production systems offer the potential of cumulative and permanent improvements (Pickering et al., 2015; de Haas et al., 2017; Beauchemin et al., 2020), setting it apart from nutritional or management strategies that often require ongoing investment or changes to farm management practices. A successful breeding program, however, is conditional on the correct choice of trait for inclusion in the breeding objective; understanding the characteristics of different candidate traits as well as the correlations among them, can help making the decision on choice of trait for inclusion. Several traits in growing cattle that consider enteric methane production as part of their calculations have been proposed including total methane (daily) production (Pickering et al., 2015), methane intensity defined as methane production per unit animal product (Pickering et al., 2013), methane yield defined as methane production per unit of feed intake (Herd et al., 2014) and residual methane defined as methane production adjusted for feed intake and liveweight (Ryan et al., 2022). No consensus currently exists as to the best trait, if any, to reflect the efficiency of methane production. In fact, it is unlikely that any one trait suits all breeding objectives (Richardson et al., 2021) but understanding the nuances, especially the shortcomings of the different methane trait definitions, is important to help make more informed decisions for both mitigation and profitability.

The main objective of the present study was to explore alternative trait definitions reflecting animal-level differences in enteric methane production and understand not just the inter-relationships among these traits, but also their associations with performance traits in growing cattle. Results from this study may help in determining the most appropriate definition of methane efficiency in growing cattle as well as the animal-level factors associated with same. Of particular interest was the inter-animal variability in these traits.

## Materials and Methods

Animal Care and Use Committee approval was not required for this study as all data were sourced from a preexisting database controlled by the Irish Cattle Breeding Federation (http://www.icbf.com).

### Data

Methane and carbon dioxide flux measurements were available from 1,601 growing beef cattle between the years 2018 and 2023, inclusive. The data consisted of 207 bulls, 435 heifers, and 959 steers. All cattle were the progeny of artificial insemination bulls and underwent performance evaluation at the Irish Cattle Breeding Federation Progeny Test Centre located in Tully, Co. Kildare, Ireland. These genetically diverse animals were sourced from commercial farms and weighed on arrival at the test center. Ancestry and breed were confirmed and corrected using array based genotypes of all animals. The genetic makeup of these animals included crosses of 13 different breeds: Aberdeen Angus, Aubrac, Belgian Blue, Charolais, Hereford, Holstein Friesian, Jersey, Limousin, Montbéliarde, Norwegian Red, Salers, Shorthorn, and Simmental. Animals entered the test station in unisex batches, hereafter referred to as groups and were grouped into pens accommodating up to 30 animals based on liveweight, breed, and sex. The average age of the animals on arrival to the test center varied by animal type, with heifers being on average 15 mo old, steers being 20 mo old, and bulls being, on average, 13 mo old. In total, there were 43 groups with an average group size of 37.2 animals.

Each pen was equipped with one Greenfeed emission monitoring system (Greenfeed, C-Lock Inc., Rapid City, SD) to record individual animal methane and carbon dioxide flux production. A total of 11 Greenfeed emission monitoring systems were used in the current study. The operational procedures and calibrations of the Greenfeed emission monitoring system have been explained elsewhere (Hristov et al., 2015; Huhtanen et al., 2015; Garnsworthy et al., 2019). The system calculates methane and carbon dioxide production per visit as a gas flux by measuring the increase in gas concentration relative to background levels. The system dispenses 30 g of concentrates every 30 s upon animal entry to encourage animals to spend time at the system. Based on the setting used in the performance test station, each animal could receive a maximum of 6 feed drops per visit every 4 h. Visits were considered complete when the animal exited the Greenfeed emission monitoring system and its radio frequency identification device tag was out of range. The concentrate used to attract animals to the system in the test station was the same concentrate included in their TMR as detailed later. Each pen also had 10 automatic Insentec feed stations (RIC Feed-Weigh Trough; Hokofarm Group BV, Marknesse, The Netherlands). The Greenfeed system and automatic feed stations both tracked animal visitations using their radio frequency identification device tags.

Following arrival at the test center, each animal underwent an acclimatization period to adjust to both the Greenfeed and Insentec recording systems, the diet and their new surroundings. Each animal was considered acclimatized to the Greenfeed system when it used the system for 3 consecutive days. To prevent interference from other animals during gas measurements, the Greenfeed system has a side gate attached that ensured that only one animal was in the measurement area at any given time. The side gate was omitted during the acclimatization period to encourage visitation. Once each animal in a pen was acclimatized to the Greenfeed machine, the side gate was reinstated. Acclimatization to the Insentec feed stations lasted between 21 and 30 d after which the feed intake measurement period commenced.

Animals were subject to multiple measurements throughout their stay at the test center, with all animals slaughtered at the end of the test, on average 96 d after entering the test station. All animals within each group were slaughtered within a week of each other at the end of their test period. Only data from the final 50 d of test were retained for use in the present study, with the exclusion of the liveweight data where a longer period was allowed. Data from 196 animals were removed as they were not in the test station for at least 50 d after the acclimatization period to the Greenfeed system.

### Diet

Heifers and steers were fed a total mixed ration (**TMR**) consisting of 13.95% hay, 40.00% water and 45.35% concentrates. It was assumed that the TMR had a dry matter content of 51% and a metabolizable energy content of 12.1 MJ/kg DM. The chemical composition of the feed has been previously described elsewhere (Smith et al., 2021). The TMR was fed ad libitum once per day using a paddle mixer wagon into the Insentec feed stations. The Insentec feed stations were filled between 0900 and 1100 from Monday to Friday and between 0830 and 1300 on Saturdays and Sundays, while the Greenfeed systems concentrate storage bins were filled every second day (or when needed).

Young bulls were fed 2 different diets: ad libitum concentrate prior to the year 2022 and TMR from 2022 on. Prior to the year 2022, young bulls started on 5 kg of concentrates per day having entered the test station. During the acclimatization period, the concentrate feeding level was increased by 0.5 kg/d until ad libitum levels were reached. A daily fixed rate of 2 kg fresh weight hay was also fed to young bulls to maintain rumen health while in the test station. Concentrates had a dry matter content of 86% and metabolizable energy content of 14.1 MJ/kg DM. It was assumed that the hay had a dry matter content of 86% and a metabolizable energy content of 8.6 MJ/kg DM. From the year 2022 onwards, all young bulls were fed the same TMR based diet as that fed to heifers and steers. Dry matter intake was defined as the kilogram of dry matter consumed by each animal per day based on the sum of their ingested feed from both the Greenfeed machine and Insentec feeders combined. Total metabolizable energy intake per animal was calculated by multiplying the daily dry matter intake of the animal by the energy content of the respective feed consumed.

### Enteric methane and carbon dioxide

Individual animal spot measurements from the Greenfeed machine underwent an initial quality control process conducted by C-Lock Inc. (C-Lock Inc., Rapid City, South Dakota), the technology provider. This process involved excluding all visits that lasted less than 2 min. In a preliminary data quality control step, visits with the lowest and highest 1% for methane production were discarded; 1,230 animals had methane flux measures in the final 50-d test period.

McGinn et al. (2021) previously validated the accuracy of Greenfeed systems for measuring methane emissions from cattle by comparing the daily methane measurements from Greenfeeds against known emissions released by a mass flow controller and a respiration chamber. Methane emissions measured by the Greenfeed were, on average, 1% higher than those from the respiration chamber, with measured emissions of 309 and 305 g/d, respectively. Ma et al. (2024) evaluated Greenfeed systems against respiration chambers in dairy cows, reporting a strong correlation of 0.84 between both measurement techniques for daily methane production. Based on the study of Arthur et al. (2018) on 616 angus cattle, a minimum of 30 visits to the Greenfeed system, each >3 min in length, is needed to generate an accurate phenotype for methane production for individual animals. In line with this recommendation, any animal that did not have at least 30 methane flux measures, each longer than 3 min, were removed. Daily visit frequency ranged from 1.8 daily visits for the bulls on a concentrate diet to 3.2 for the heifers. An additional restriction of requiring at least one methane flux measure within 10 d of test end was included in the present study. A total of 216 animals that did not meet these criteria during the final 50-d test period and were therefore excluded from further consideration.

Methane flux and carbon dioxide flux was defined as the flux measurement recorded by the Greenfeed machine measured in grams per day. As an animal can have multiple flux measures each day, methane production was calculated as the average of all methane flux measures over the test period for each animal, yielding a single value in grams of methane per day (i.e., g CH_4_/d). Similarly average carbon dioxide production was calculated as the average of all carbon dioxide flux measures over the test period for each animal (i.e., g CO_2_/d).

### Liveweight and average daily gain

Animals were weighed, on average, every 3 wk. Only animals with at least 3 liveweight records after entering the test station were included in the calculation of average daily gain (**ADG**) and metabolic liveweight; 46 animals had less than 3 liveweight records so were not considered further in any analysis. Simple linear regression was fitted through the serial liveweight measures of each individual animal separately to estimate a model intercept and ADG (i.e., linear regression coefficient) per animal; the same approach was also fitted through the serial measures of metabolic liveweight to be subsequently used in the calculation of mid-test metabolic liveweight. A total of 5 animals where the coefficient of determination of the linear regression of liveweight on days of test was <90% were not considered further. Mid-test liveweight (herein referred to as just liveweight) was defined as the expected liveweight 25 d before the end of the test derived from the model intercept and linear regression coefficient of liveweight measures on days of test for each animal separately; mid-test metabolic liveweight was calculated using the same approach but where the dependent variable was metabolic liveweight. A further 24 animals over 30 mo of age at the end of test were removed from all analysis; all other animals were between 14 and 27 mo of age at slaughter. Following all edits, ADG, liveweight, mid-test metabolic liveweight, feed intake, carbon dioxide and methane emission data, were available on 939 animals consisting of 73 bulls fed TMR, 65 bulls fed concentrates, 320 heifers, and 481 steers.

### Ultrasound measures

Ultrasound measurements on live animals were available for 642 of the animals with methane data all recorded within 10 d of slaughter. The ultrasound measurements were eye muscle depth (**MD**; mm), fat depth (**FD**; mm), and intramuscular fat (IMF; %). Ultrasound measurements were recorded using an Esaote-Pie Medical Aquila PRO Vet ultrasound scanner with a 3.5 MHZ transducer head. Eye muscle depth was recorded at the third lumbar vertebra. The measurement was taken on top of the loin that had the deepest part of the muscle. Fat depth was recorded in 2 areas; the 13th thoracic rib in 4 locations 2 cm apart and the third lumbar vertebrae in 3 locations 2 cm apart. Intramuscular fat was recorded at the 13th thoracic rib.

### Muscular and skeletal traits

Muscular and skeletal traits were subjectively scored at the test station on 598 of the test animals when alive. Muscular traits were recorded on a scale of 1 (poor muscling) to 15 (excellent muscling) (Doyle et al., 2018). These traits were width at withers, width behind withers, loin development, development of hind quarter, and thigh width. The skeletal traits were measured on a scale of 1 (small) to 10 (large). These traits were chest depth, chest width, height of withers, length of back, pelvic length, and width at hips. All linear traits were scored by the same trained assessor.

### Carcass characteristics

All animals were slaughtered, on average, 3 d after completing the test period. All animals were slaughtered at a single commercial meat abattoir located 73.5 km from the testing facility. Carcass data were available for 939 animals that also had methane emission data. Animals were processed for slaughter within an hour of arrival at the facility, and the carcass weight was recorded within 1 h after slaughter. Both carcass conformation and carcass fat cover were assessed on a 15-point scale using video imaging analysis equipment (Pabiou et al., 2011). A carcass conformation score of 1 represented poor conformation while a score of 15 reflected excellent conformation (Englishby et al., 2016). A carcass fat score of 1 signified low fat cover while 15 represented high fat cover.

### Feed behavior traits

Individual animal feed intake measures were available from the Insentec feed stations that recorded every 100 g fluctuation in feed removed from the feed bin. It was assumed that feed disappearance from the feed bin was equivalent to intake. The feed stations also recorded the duration of each feed event. There was no minimum length for feed events but feed events with a duration of greater than 60 min were considered errors and removed. All feed events for animals in the affected pen on those days in which the errors occurred were subsequently removed. Such long feed events likely happened when the automatic feed station did not record the animal exiting or failed to register another animal entering the feed station immediately after (Kelly et al., 2020). A total of 2,538 individual feed records (0.16% of the data) were removed due to this edit. Eating rate per feed event was defined as the metabolizable energy intake for an individual animal for that feed event divided by the length of the feed event in minutes. Eating rate was only calculated using the feed consumed from the Insentec. Eating time per day was defined as the amount of time spent eating each day in minutes.

### Adjustment for management group effects

Because of the system operated at the test station, management group was completely confounded with sex hindering the ability to disentangle both effects. Therefore, all traits were firstly adjusted for the management group effect using a mixed model in SAS (PROC MIXED). The fitted statistical model used was;


$$\begin{array}{l} y\;=\;sex+\underset{j=1}{\overset{13}{\sum }}\;Breed_{j}+GROUP+e \end{array}$$

where *y* is the dependant variable of methane production (average of all methane flux measures over the test period for each animal), carbon dioxide production, energy intake, liveweight, carcass weight, metabolic liveweight, ADG, carcass fat, carcass conformation, fat depth, muscle depth, intramuscular fat, feed behavior traits, skeletal and muscular traits; sex is the fixed effect of sex (i.e., heifer, steer, bulls fed concentrates or bulls fed TMR); $\underset{j=1}{\overset{13}{\sum }}\;Breed_{j}$ is the fixed effects of the animal’s breed proportion for each of the 13 breeds (Table 1); group is the random effect for the management group; and *e* is the residual term. The estimated group effects were subsequently subtracted from the dependent variables, and these adjusted traits were used in all subsequent analyses.

**Table 1.** Raw means (standard deviation in parentheses) of the Greenfeed system measurements, feed intake and behavior measurements, performance traits, ratio traits and residual traits of steers, heifers, bulls on a TMR diet and bulls on a concentrate diet

	Steer	Heifer	Bull TMR	Bull concentrates
No. Animals	481	320	73	65
Final age, d	694.46 (79.06)	541.32 (53.93)	502.9 (22.06)	495.51 (25.09)
Number of Greenfeed visits	141.91 (39.28)	155.10 (35.30)	105.49 (32.20)	71.05 (23.02)
Methane, g/d	255.92 (41.45)	235.20 (35.25)	262.21 (42.16)	143.35 (35.01)
Carbon dioxide, g/d	10,163.3 (952.8)	8,895.3 (845.2)	10,350.3 (940.6)	9,652.8 (1,010.2)
ADG^1^, kg/d	1.38 (0.24)	1.43 (0.24)	2.10 (0.29)	1.98 (0.34)
Energy intake, ME/d	154.96 (16.90)	137.69 (16.62)	142.45 (15.70)	172.46 (21.34)
Liveweight, kg	623.72 (51.47)	529.66 (48.73)	621.35 (62.14)	631.17 (55.88)
Carcass weight, kg	349.31 (34.52)	308.79 (30.87)	401.21 (43.70)	397.78 (39.74)
Carcass fat, scale 1 to 15^2^	8.90 (1.49)	9.36 (2.07)	6.72 (1.23)	7.15 (1.90)
Carcass conformation, scale 1 to 15^2^	6.55 (3.04)	8.81 (2.09)	10.53 (1.83)	10.62 (2.29)
Fat depth, mm	5.37 (1.52)	5.73 (1.76)	3.58 (0.77)	3.99 (1.29)
Muscle depth, mm	67.22 (11.95)	73.68 (7.17)	81.55 (6.39)	82.72 (7.77)
Intramuscular fat, %	7.10 (0.85)	6.73 (1.14)	6.22 (0.92)	6.00 (1.06)
Methane yield, g CH4/MJ	1.66 (0.24)	1.71 (0.20)	1.85 (0.27)	0.83 (0.18)
Methane yield, g CH4/kg DMI	20.09 (2.91)	20.79 (2.37)	22.44 (3.21)	11.21 (2.38)
CH_4_:CO_2_ ratio, g CH_4_/g CO_2_	0.025 (0.003)	0.026 (0.003)	0.025 (0.003)	0.015 (0.003)
MIL^1^, g CH_4_/kg lw	0.41 (0.06)	0.44 (0.06)	0.42 (0.06)	0.23 (0.05)
MIC^1^, g CH_4_/kg cw	0.74 (0.13)	0.77 (0.12)	0.66 (0.10)	0.36 (0.10)
MADG^1^, g CH_4_/kg ADG	190.33 (42.18)	167.65 (29.73)	127.58 (29.07)	73.65 (18.37)
Eating time/d, min	118.99 (20.31)	134.3 (21.60)	116.47 (19.27)	70.59 (20.84)
Energy eat rate, MJ/min	1.29 (0.23)	1.00 (0.16)	1.22 (0.26)	4.12 (0.95)^3^
Daily feed events	28.84 (7.95)	33.85 (8.90)	25.77 (7.33)	32.56 (6.77)
MEI^1^ per feed event	5.55 (1.47)	4.16 (1.18)	5.80 (1.75)	5.38 (1.10)
RMP_lw^1^_, g/d	68.07 (38.14)	75.68 (31.00)	75.06 (34.31)	—
RMP_cw^1^_, g/d	124.78 (40.75)	119.43 (34.39)	111.80 (36.78)	—
RMP_adg^1^_, g/d	191.73 (39.76)	160.29 (31.40)	270.78 (42.14)	—
RMP_energy^1^_, g/d	72.11 (36.13)	71.88 (26.56)	93.83 (35.79)	—
RMP_el^1^_, g/d	51.39 (36.05)	55.55 (26.79)	70.68 (34.74)	—
RMP_la^1^_, g/d	66.44 (37.92)	54.23 (29.52)	128.86 (34.63)	—
RMP_ca^1^_, g/d	113.87 (39.99)	91.14 (31.76)	180.54 (36.83)	—

^1^ADG, average daily gain; MIL, methane intensity liveweight; MIC, methane intensity carcass weight; MADG, methane intensity average daily gain; lw, liveweight; cw, carcass weight; MEI, metabolizable energy intake; RMP_lw_, methane adjusted for liveweight; RMP_cw_, methane adjusted for carcass weight; RMP_adg_, methane adjusted for average daily gain; RMP_energy_, methane adjusted for energy intake; RMP_el_, methane adjusted for energy intake and liveweight; RMP_el_, methane adjusted for energy intake and liveweight; RMP_la_, methane adjusted for liveweight and average daily gain; RMP_ca_, methane adjusted for carcass weight and average daily gain. Residual traits were not calculated for bulls on a concentrate diet.

^2^Scale of 1 to 15. A score of 1 represents poor conformation or a lean carcass, and a score of 15 represents a well conformed or fat carcass.

^3^Energy eat rate for bulls on a concentrate diet was for the concentrates in the Insentec feeders only.

### Ratio traits

Five ratio traits were generated as a measure of methane efficiency.

Methane yield for each animal was calculated as


$$\begin{array}{l} MY=\frac{Methane\;production}{Metabolisable\;energy\;intake} \end{array}$$

Methane intensity for each animal was defined in 3 ways to consider the average methane produced in grams 1) per kilogram of liveweight, 2) per kilogram of carcass weight, and 3) per kilogram of ADG.


$$\begin{array}{llll} & \;\;MIL=\frac{Methane\;\;\;production}{Liveweight}\; & \;\;MIC=\frac{Methane\;\;\;production}{Carcass\;weight}\; & MADG=\frac{Methane\;\;\;production\;}{ADG} \end{array}$$

where methane production is the average methane production, liveweight is the animal’s mid-test liveweight in kilograms, carcass weight is the animal’s carcass weight in kilograms, and ADG is the animal’s ADG in kilograms per day.

Methane to carbon dioxide ratio was defined as


$$\begin{array}{l} CH_{4}:CO_{2}=\frac{Methane\;production}{Carbon\;dioxide\;production} \end{array}$$

### Residual traits

A series of residual methane traits were estimated to reflect the methane production adjusted for performance phenotypes of ADG, liveweight, carcass weight, and energy intake. The modeling of methane production was progressively built up with different combinations of the performance traits as independent variables based on their association (*P* < 0.05) with actual methane output; all analyses were undertaken using linear fixed effects models in SAS (SAS Institute Inc., Cary, NC). Bulls-fed concentrates were not included in these analyses due to a low number of records. The fixed effects of sex (heifer, steer, or bull fed TMR) and 13 breed covariates were included in all models; neither dam parity, recombination loss, and heterosis were associated with methane output so were therefore not considered. In each model, interactions between the variables with sex were initially tested for. Only statistically significant (*P* < 0.05) interactions were included. No non-linear associations with the performance traits were detected (*P* > 0.05). The model used was


$$\begin{array}{l} y_{ijklmn}=sex_{j}+\underset{k=1}{\overset{13}{\sum }}\;Breed_{k}+variable_{l}+variable_{m}+variable_{n}+e_{ijklmn} \end{array}$$

where *y*_*ijklmn*_ is the methane production of animal *i*; sex_*j*_ was the fixed effect of the *j*th sex of animal *i* (heifer, steer, or bulls-fed TMR); $\underset{k=1}{\overset{13}{\sum }}\;Breed_{k}$ is the fixed effects of the animals breed proportion for each of the 13 breeds; variable_*l*_ is one of liveweight, energy intake, ADG, carcass weight; variable_*m*_ is of the subset null, liveweight, energy intake, ADG or carcass weight where variable_*m*_ ≠ variable_*n*_ or variable_*l*_; variable_*n*_ is of the subset null, liveweight, energy intake, ADG or carcass weight where variable_*n*_ ≠ variable_*m*_ or variable_*l*_. In total, 7 different residual methane traits were generated (Table 2) for 874 animals. These traits were methane production adjusted for just liveweight (RMP_lw_), methane production adjusted for just carcass weight (RMP_cw_), methane production adjusted for just ADG (RMP_adg_), methane production adjusted for just energy intake (RMP_energy_), methane production adjusted for both energy intake and liveweight (RMP_el_), methane production adjusted for both carcass weight and ADG (RMP_ca_), and methane adjusted for all of energy intake and liveweight and ADG (RMP_la_). The number of animals with recorded measures for each trait is shown in Supplementary Table 1.

**Table 2.** The partial regression coefficients (standard error in parenthesis) for a series of different models regressing average methane production over the test period on a series of different performance traits for steers, heifers and bulls on a TMR diet, with the coefficient of determination (RMSE in parenthesis)

Energy intake	Liveweight	Carcass weight	Average daily gain			*R* ^2^ (RMSE g CH_4_/d)^1^
			Steer	Heifer	Bull	
1.19 (0.07)						0.422 (31.24)
	0.30 (0.02)					0.372 (32.56)
		28.49 (7.71)				0.326 (33.62)
			46.57 (7.39)^a^	52.41 (8.41)^a^	−4.08 (14.10)^b NS^	0.308 (34.21)
0.98 (0.11)	0.08 (0.03)					0.426 (31.13)
	0.26 (0.03)		19.07 (7.45)^a^	29.65 (8.23)^a^	−13.92 (13.30)^b NS^	0.385 (32.26)
		0.29 (0.04)	28.49 (7.71)^a^	37.33 (8.39)^a^	−17.24 (14.10)^b NS^	0.351 (33.05)
1.17 (0.10)		1.20 (0.11)^NS^				0.424 (31.10)
1.23 (0.09)			−4.65 (5.92)^NS^			0.427 (31.16)
1.02 (0.12)	0.08 (0.03)		−4.21 (5.91)^NS^			0.431 (31.06)
1.21 (0.11)		0.01 (0.05)^NS^	−4.98 (6.00)^NS^			0.429 (31.01)

^1^ *R* ^2^, coefficient of determination.

^a,b,c^Regressions with different superscripts in each row for each trait were significantly different (*P* < 0.05).

^NS^Independent variable was not related to the dependent variable in this row.

A linear fixed effects model was also used to explore the associations between both animal sex and breed with the ratio-based methane traits and other performance traits. Associations among all traits were estimated through partial correlations, adjusted for animal sex and breed using the MANOVA/PRINTE statement within the GLM procedure of SAS. Correlation coefficients were classified as strong (*r* > 0.6), moderate (*r* between 0.4 and 0.6), or weak (*r* < 0.4), respectively.

## Results

Raw summary statistics for the range of traits across sexes (including bulls fed concentrates) are in Table 1; distinct differences in methane production, growth rate, and feed efficiency existed by sex and, within bulls, diet. Bulls on the concentrate diet emitted the least methane (143 g/d), despite being the heaviest and eating the most. Bulls on the concentrate diet also had lower values for each of the methane ratio traits. Bulls on the concentrate diet also had the fastest eating rate and shorter daily eating duration compared to the bulls on the TMR diet. However, as well as being on a different diet, bulls on a concentrate diet were measured in a different set of years compared to bulls on the TMR diet.

### Associations between performance traits and daily methane production

The partial regression coefficients of methane production on energy intake, liveweight, carcass weight, and ADG either individually or as different combinations are presented in Table 2 for animals just fed TMR. ADG was the only trait where the association with daily methane production differed (*P* < 0.05) by animal sex. ADG on its own was positively associated with methane production in both heifers and steers, but there was no relationship between ADG and methane production in bulls fed TMR.

Breed and animal sex combined explained 24.7% of the variation in daily methane production with a model root mean square error (**RMSE**) of 35.62 g/d. Including also ADG in the model resulted in the least improvements to the coefficient of determination of methane production (*R*^2^ = 0.31). Liveweight and ADG together in the model (along with sex and breed) explained 38.5% of the variation in methane production. Once energy intake was included in the model, neither carcass weight nor ADG was associated with methane production. Including just liveweight or energy intake in the model (along with sex and breed) resulted in an *R*^2^ of 0.37 and 0.42, respectively. Including any ultrasound or carcass fat measures in the model did not improve (*P* > 0.05) the fit to the data. Meanwhile, including both liveweight and energy intake in the model for methane production resulted in a *R*^2^ of 0.43, with a RMSE of 31.13 g/d. The difference in the mean of the residuals between the top and bottom 10% of animals ranked on RPM_el_ was 109.44 g/d indicating substantial variability in methane production even after adjustments for liveweight and intake.

### Sex effects

The predicted marginal means for the range of methane traits investigated are summarized in Table 3 for heifers, steers, and bulls fed TMR; bulls on concentrates were not included due to the low number of records. Of all the residual methane traits, carcass weight-adjusted residual methane (RMP_cw_) was the only trait where no sex differences existed. Bulls on a TMR diet produced the most methane per day of 269.53 g (SE = 4.48 g), while heifers and steers produced 237.54 g (SE = 2.22 g) and 253.26 g (SE = 1.84 g), respectively. Irrespective of how residual methane was defined, bulls consistently had greater residual methane values compared to both heifers and steers with the exception of methane adjusted for either liveweight (RMP_lw_) or carcass weight (RMP_cw_). Generally, no difference between heifers and steers existed for the residual methane traits; the exception was RMP_adg_ and RMP_ca_ where all 3 sexes differed from one another, and RMP_lw_ where there was no difference between heifers and bulls, but steers had a lower mean.

**Table 3.** Predicted marginal means (standard error in parenthesis) of methane production and multiple residual methane traits for steers, heifers, and bulls on a TMR diet

Trait^1^	Steer	Heifer	Bull TMR
Methane	253.26 (1.84)^a^	237.54 (2.22)^b^	269.53 (4.48)^c^
RMP_adg_	187.83 (1.76)^a^	164.28 (2.13)^b^	279.00 (4.30)^c^
RMP_cw_	119.2 (1.75)^a^	125.11 (2.10)^a^	124.24 (4.51)^a^
RMP_lw_	65.17 (1.68)^a^	78.43 (2.03)^b^	82.10 (4.09)^b^
RMP_energy_	72.15 (1.61)^a^	71.81 (1.95)^a^	93.82 (3.95)^b^
RMP_ca_	108.17 (1.71)^a^	97.03 (2.06)^b^	192.85 (4.42)^c^
RMP_la_	63.09 (1.66)^a^	57.57 (2.01)^a^	136.31 (4.05)^b^
RMP_el_	50.90 (1.60)^a^	56.03 (1.94)^a^	71.85 (3.94)^b^

^1^Methane, average methane production adjusted for breed; RMP_adg_, methane adjusted for average daily gain; RMP_cw_, methane adjusted for carcass weight; RMP_lw_, methane adjusted for liveweight; RMP_energy_, methane adjusted for energy intake; RMP_ca_, methane adjusted for carcass weight and average daily gain; RMP_la_, methane adjusted for liveweight and average daily gain; RMP_el_, methane adjusted for energy intake and liveweight.

^a,b,c^Least squares means within a row with different subscripts differ (*P* < 0.05).

### Breed effects

The partial regression coefficients of the methane traits on the proportion of each breed, relative to the Limousin breed are in Table 4. Limousins, on average, produced the least amount of methane of all the breeds investigated. Herefords and Montbéliarde produced 124.50 and 130.77 g more methane per day, respectively, than Limousins. Of the residual methane traits, Herefords, Montbéliardes, and Shorthorns consistently produced the most methane relative to Limousins, while Norwegian Reds and Belgian Blues consistently had the closest residual methane production values to Limousins. The ranking of breeds based on residual methane production did not stay constant across the residual methane trait definitions, suggesting different traits may explain methane production differently across breeds.

**Table 4.** Model regression coefficients (SE in parenthesis, g CH_4_/d), relative to a Limousin, from regressing methane production on animal breed composition^1^ with and without adjusting for a series of different performance traits^2^

	Methane	RMP_adg_	RMP_cw_	RMP_lw_	RMP_energy_	RMP_ca_	RMP_la_	RMP_el_
HF	49.96 (6.32)	49.17 (6.06)	58.55 (6.02)	38.63 (5.77)	17.58 (5.54)	55.48 (5.91)	39.08 (5.71)	20.06 (5.52)
AA	35.89 (7.58)	24.50 (7.26)	43.85 (7.18)	29.77 (6.92)	3.79 (6.64)	34.14 (7.05)	24.64 (6.85)	7.68 (6.62)
AU	33.00 (10.86)	38.81 (10.41)	34.54 (10.75)	33.73 (9.92)	31.93 (9.52)	37.49 (10.55)	36.50 (9.81)	32.30 (9.48)
BB	25.76 (16.33)	32.10 (15.66)	8.25 (15.52)	8.23 (14.92)	13.35 (14.32)	16.48 (15.23)	14.31 (14.76)	10.57 (14.26)
CH	41.16 (8.23)	40.18 (7.89)	31.16 (7.80)	23.19 (7.51)	23.88 (7.21)	31.53 (7.65)	24.14 (7.43)	21.88 (7.19)
HE	124.53 (10.50)	108.14 (10.07)	126.00 (9.93)	107.16 (9.59)	83.39 (9.21)	114.68 (9.74)	101.27 (9.49)	85.69 (9.17)
JE	18.21 (9.80)	36.51 (9.39)	47.22 (9.27)	32.68 (8.95)	21.58 (8.59)	51.32 (9.10)	37.51 (8.85)	25.06 (8.55)
MO	130.77 (68.74)	140.26 (65.91)	118.39 (73.72)	151.42 (62.79)	111.84 (60.25)	124.37 (72.35)	149.59 (62.11)	120.93 (60.02)
NR	22.20 (39.84)	14.78 (38.19)	21.24 (37.61)	−9.59 (36.39)	−15.72 (34.92)	15.74 (36.9)	−9.93 (36.00)	−18.04 (34.78)
SA	23.99 (11.80)	27.73 (11.32)	34.71 (11.16)	27.31 (10.78)	32.71 (10.35)	32.43 (10.95)	26.99 (10.67)	32.09 (10.31)
SH	57.13 (14.19)	45.63 (13.61)	61.32 (13.40)	47.88 (12.96)	35.19 (12.44)	51.13 (13.15)	42.30 (12.82)	36.39 (12.39)
SI	61.83 (9.96)	50.29 (9.55)	55.88 (9.40)	41.07 (9.10)	27.73 (8.73)	49.14 (9.23)	37.66 (9.00)	27.85 (8.70)

^1^HF, Holstein Friesian; AA, Aberdeen Angus; AU, Aubrac; BB, Belgian Blue; CH, Charolais; HE, Hereford; JE, Jersey; MO, Montbéliarde; NR, Norwegian Red; SA, Salers; SH, Shorthorn; SI, Simmental.

^2^Methane, average methane production; RMP_adg_, methane adjusted for average daily gain; RMP_cw_, methane adjusted for carcass weight; RMP_lw,_ methane adjusted for liveweight; RMP_energy_, methane adjusted for energy intake; RMP_ca_, methane adjusted for carcass weight and average daily gain; RMP_la_, methane adjusted for liveweight and average daily gain; RMP_el_, methane adjusted for energy intake and liveweight.

### Correlations

The partial correlations among the traits investigated, having adjusted for animal sex and breed, are in Tables 5 and 6. Larger framed animals, as reflected by higher skeletal scores, on average, emitted more methane daily with the strength of the correlations varying from 0.24 (i.e., length of back) to 0.33 (i.e., depth of chest) between each skeletal trait and methane production (Table 6). This is consistent with the observed positive correlations between daily methane production and both liveweight (0.41, Table 5) and carcass weight (0.32, Table 5), again indicating that larger animals, on average, produce more methane. An animal’s muscular conformation, as reflected by muscular scores and carcass conformation, was not associated with methane production, with the exception of thigh width which had a 0.14 positive correlation with methane output indicating that animals with more muscle were associated with more methane emitted daily. This is consistent with the observed positive correlation (0.15) between daily methane production and muscle depth measured using ultrasound on live animals (Table 5). Feeding behavior traits were associated with methane production having adjusted for animal sex, breed, and energy intake (Table 7). Methane production was positively associated with both eating rate (0.09) and intake per feed event (0.11), while being negatively associated with eating time (−0.09) and number of daily feed events (−0.10) among animals consuming the same amount of energy.

**Table 5.** Partial correlations among and between methane production, carbon dioxide production, energy intake, average daily gain, liveweight and carcass weight, carcass conformation, carcass fat, MD, FD and intramuscular fat after adjusting for breed and animal type (steer, heifer or bull on TMR diet)

	Methane	Carbon dioxide	Energy intake	ADG^1^	Liveweight	Carcass weight	Carcass conformation	Carcass Fat	Muscle depth	Fat depth
Carbon dioxide	0.58									
Energy intake	0.48	0.73								
Average daily gain	0.26	0.50	0.58							
Liveweight	0.41	0.68	0.73	0.41						
Carcass weight	0.32	0.62	0.65	0.40	0.93					
Carcass conformation	0.04^*^	0.17	0.15	0.10	0.23	0.29				
Carcass fat^2^	0.19	0.26	0.35	0.16	0.39	0.33	0.10			
Muscle depth	0.15	0.28	0.30	0.22	0.41	0.48	0.34	0.22		
Fat depth	0.16	0.18	0.31	0.15	0.30	0.24	0.08^*^	0.53	0.12	
Intramuscular fat	0.18	0.15	0.14	0.05^*^	0.14	0.08^*^	0.06^*^	0.24	0.05^*^	0.29

^1^ADG, average daily gain.

^2^Scale of 1 to 15: a score of 1 represents poor conformation or a lean carcass, and a score of 15 represents a well conformed or fat carcass.

^*^ *P* ≥ 0.05.

**Table 6.** Partial correlations between methane production, carbon dioxide production, energy intake, average daily gain (ADG), liveweight and carcass weight with the methane ratio traits, residual traits and the skeletal and muscular traits after adjusting for breed and animal type (steer, heifer, or bull on TMR diet)

	Methane	Carbon dioxide	Energy intake	ADG	Liveweight	Carcass weight
Methane yield	0.74	0.08	−0.22	−0.15	−0.11	−0.15
MIL^1^	0.82	0.20	0.07^*^	0.03^*^	−0.18	−0.24
MIC^1^	0.80	0.19	0.08	0.01^*^	−0.17	−0.30
MADG^1^	0.51	0.01^*^	−0.15	−0.63	−0.06^*^	−0.12
CH_4_:CO_2_ ratio	0.79	−0.04^*^	0.05^*^	−0.05^*^	−0.01^*^	−0.08
RMP_adg^1^_	0.96	0.47	0.33	0.00^*^	0.30	0.22
RMP_cw^1^_	0.95	0.40	0.29	0.14	0.12	0.00^*^
RMP_lw^1^_	0.91	0.33	0.20	0.10	0.00^*^	−0.07
RMP_energy^1^_	0.88	0.26	0.00^*^	−0.02^*^	0.06^*^	0.00^*^
RMP_ca^1^_	0.93	0.37	0.23	0.00^*^	0.11	0.00^*^
RMP_la^1^_	0.90	0.31	0.16	0.00^*^	0.00^*^	−0.07
RMP_el^1^_	0.87	0.25	0.00^*^	−0.02^*^	0.00^*^	−0.06^*^
Width at withers^2^	0.04^*^	0.12	0.11	0.02^*^	0.27	0.35
Width behind withers^2^	0.05^*^	0.11	0.13	0.02^*^	0.28	0.37
Loin development^2^	0.08^*^	0.15	0.15	0.03^*^	0.31	0.38
Hind quarter^2^	−0.04^*^	0.08^*^	0.03^*^	−0.03^*^	0.16	0.28
Thigh width^2^	0.14	0.29	0.24	0.07^*^	0.46	0.53
Depth of chest^3^	0.33	0.42	0.45	0.23	0.57	0.52
Width of chest^3^	0.32	0.38	0.39	0.20	0.57	0.56
Height of withers^3^	0.28	0.47	0.48	0.26	0.60	0.55
Length of back^3^	0.24	0.42	0.42	0.22	0.57	0.53
Pelvic length^3^	0.30	0.43	0.50	0.25	0.58	0.53
Width at hips^3^	0.27	0.36	0.41	0.20	0.54	0.49

^1^MIL, methane intensity liveweight; MIC, methane intensity carcass weight; MADG, methane intensity average daily gain; RMP_energy_, methane adjusted for energy intake; RMP_adg_, methane adjusted for average daily gain; RMP_lw_, methane adjusted for liveweight; RMP_cw_, methane adjusted for carcass weight; RMP_el_, methane adjusted for energy intake and liveweight; RMP_la_, methane adjusted for liveweight and average daily gain; RMP_ca_, methane adjusted for carcass weight and average daily gain.

^2^Scale of 1 to 15. A score of 1 represents poor muscling, and a score of 15 represents excellent muscling.

^3^Scale of 1 (small) to 10 (large).

^*^ *P* > 0.05.

**Table 7.** Partial correlations between methane production, carbon dioxide production, carcass weight and average daily gain with eating rate, intake per feed event, eating time and number of daily feed events adjusting for animal type (steer, heifer, or bull on TMR diet), energy intake and liveweight

	Methane^1^	Carbon dioxide^1^	Carcass weight	Average daily gain
Eating rate	0.085	−0.007^*^	−0.026^*^	−0.121
Intake per event	0.114	−0.003^*^	−0.006^*^	−0.043^*^
Eating time	−0.085	−0.008^*^	0.012^*^	0.091
Daily feed events	−0.097	−0.022^*^	0.005^*^	0.004^*^

^1^Methane = methane production, carbon dioxide = carbon dioxide production.

^*^ *P* ≥ 0.05.

The ratio traits of CH_4_:CO_2_, MY, MIL, MIC, and MADG were all positively correlated with methane production (the numerator in formula), and were either not correlated or negatively correlated with the production traits of energy intake, ADG, liveweight, and carcass weight which, when in the formulae, were in the denominator (Table 6). Methane intensity using carcass weight in the denominator and MIL had a correlation of 0.80 and 0.82 with methane production (Table 8), respectively, although neither was correlated with ADG (Table 6). Liveweight and carcass weight were strongly correlated with one another (0.93), which was also reflected in the strong correlation between MIL and MIC (0.97) (Table 8). Liveweight was more strongly correlated with methane (0.41, Table 5) than the correlation between carcass weight and methane production (0.32, Table 5).

**Table 8.** Partial correlations among and between the ratio and residual traits^1^ adjusting for breed and animal type (steer, heifer, or bull on TMR diet)

	Methane	MY	MIL	MIC	MADG	CH_4_:CO_2_	RMP_adg_	RMP_cw_	RMP_lw_	RMP_e_	RMP_ca_	RMP_la_
MY	0.74											
MIL	0.82	0.86										
MIC	0.80	0.83	0.97									
MADG	0.51	0.69	0.59	0.59								
CH_4_:CO_2_	0.79	0.84	0.86	0.84	0.62							
RMP_adg_	0.96	0.81	0.84	0.82	0.72	0.82						
RMP_cw_	0.95	0.82	0.94	0.94	0.58	0.85	0.94					
RMP_lw_	0.91	0.86	0.98	0.95	0.59	0.86	0.92	0.98				
RMP_e_	0.88	0.97	0.90	0.87	0.67	0.87	0.91	0.92	0.93			
RMP_ca_	0.93	0.85	0.93	0.93	0.70	0.86	0.97	0.98	0.97	0.93		
RMP_la_	0.90	0.87	0.97	0.94	0.68	0.87	0.94	0.97	0.99	0.94	0.99	
RMP_el_	0.87	0.96	0.93	0.90	0.67	0.88	0.91	0.94	0.96	0.99	0.95	0.96

^1^Methane, average methane production; MY, methane yield; MIL, methane intensity liveweight; MIC, methane intensity carcass weight; MADG, methane intensity average daily gain; CH_4_:CO_2_, CH_4_:CO_2_ ratio; RMP_adg_, methane adjusted for average daily gain; RMP_cw_, methane adjusted for carcass weight; RMP_lw,_ methane adjusted for liveweight; RMP_e_, methane adjusted for energy intake; RMP_ca_, methane adjusted for carcass weight and average daily gain; RMP_la_, methane adjusted for liveweight and average daily gain; RMP_el_, methane adjusted for energy intake and liveweight.

All residual traits were positively associated with both methane production (0.87 to 0.96, Table 6) and carbon dioxide production (0.25 to 0.47, Table 6). With the exception of both RMP_energy_ and RMP_el_, methane production had a correlation of at least 0.90 with each of the residual traits indicating that the ranking of these animals for these traits was similar irrespective of the definition; methane production had a correlation of 0.88 and 0.87 with RMP_energy_ and RMP_el_, respectively. The association between the residual traits with the muscular and skeletal traits varied from −0.14 to 0.27 (Table 9). Similarly, the association between the residual traits with the production traits of energy intake, ADG, liveweight, and carcass weight ranged from non-existent to positive correlations as strong as 0.33 (RMP_adg_ with energy intake). RMP_energy_, RMP_el_, and RMP_la_ were not correlated with any of the production traits. Energy intake was positively associated with RMP_adg_ (0.33), RMP_lw_ (0.20), RMP_cw_ (0.29), and RMP_ca_ (0.23), indicating that animals with lower energy intake had lower residual methane production where methane production was not adjusted for energy intake.

**Table 9.** Partial correlations between the ratio and residual traits^1^ with feeding behavior traits and muscular and skeletal traits adjusting for breed and animal type (steer, heifer, or bull on TMR diet)

	MY	MIL	MIC	MADG	CH_4_:CO_2_	RMP_adg_	RMP_cw_	RMP_lw_	RMP_e_	RMP_ca_	RMP_la_	RMP_el_
Daily feed events	−0.16	−0.01^*^	−0.02^*^	−0.10	−0.06^*^	−0.03^*^	−0.01^*^	−0.01^*^	−0.11	−0.03^*^	−0.02^*^	−0.09
Intake per event	0.08	0.05^*^	0.07^*^	0.08	0.11	0.19	0.15	0.10	0.13	0.14	0.10	0.11
Eating time	−0.15	−0.02^*^	−0.01^*^	−0.15	−0.06^*^	−0.03^*^	0.01^*^	−0.01^*^	−0.09	−0.03^*^	−0.04^*^	−0.08
Eating rate	0.00^*^	0.06^*^	0.06^*^	0.07^*^	0.09	0.24	0.16	0.13	0.09	0.17	0.13	0.08
Depth of chest^2^	0.01^*^	−0.03^*^	−0.02^*^	0.05^*^	0.08	0.27	0.16	0.09	0.13	0.15	0.09	0.09
Width of chest^2^	0.04^*^	−0.03^*^	−0.04^*^	0.07^*^	0.10	0.27	0.14	0.08^*^	0.15	0.14	0.08	0.10
Height of withers^2^	−0.08^*^	−0.11	−0.10	0.00^*^	−0.02^*^	0.22	0.09	0.02^*^	0.05^*^	0.09	0.02^*^	0.01^*^
Length of back^2^	−0.07^*^	−0.12	−0.12	0.01^*^	−0.02^*^	0.20	0.06^*^	0.00^*^	0.04^*^	0.06^*^	0.00^*^	0.00^*^
Pelvic length^2^	−0.07^*^	−0.06^*^	−0.06^*^	0.02^*^	0.04^*^	0.24	0.12	0.05^*^	0.06^*^	0.11	0.05^*^	0.03^*^
Thigh width^3^	−0.04^*^	−0.16	−0.20	0.04^*^	−0.05^*^	0.12	−0.03^*^	−0.07	0.02^*^	−0.01^*^	−0.05^*^	−0.02^*^
Loin development^3^	−0.02^*^	−0.12	−0.15	0.04^*^	−0.02^*^	0.07^*^	−0.04^*^	−0.06^*^	0.01^*^	−0.02^*^	−0.05^*^	−0.02^*^
Hind quarter^3^^,^^5^	−0.06^*^	−0.16	−0.22	0.00^*^	−0.12	−0.03^*^	−0.14	−0.13	−0.07^*^	−0.11	−0.11	−0.09
Width at withers^3^	−0.04^*^	−0.13	−0.17	0.01^*^	−0.04^*^	0.04^*^	−0.08	−0.09	−0.01^*^	−0.05^*^	−0.07^*^	−0.04^*^
Width behind withers^3^	−0.05^*^	−0.14	−0.18	0.02^*^	−0.03^*^	0.04^*^	−0.08	−0.09	−0.02^*^	−0.05^*^	−0.07^*^	−0.05^*^
Carcass conformation^4^	−0.06^*^	−0.09	−0.14	−0.05^*^	−0.08	0.02^*^	−0.05^*^	−0.06^*^	−0.03^*^	−0.05^*^	−0.05^*^	−0.05^*^
Carcass fat^4^	−0.06^*^	−0.03^*^	−0.01^*^	−0.01^*^	0.04^*^	0.15	0.09	0.04^*^	0.03^*^	0.08	0.03^*^	0.01^*^
Fat depth	−0.06^*^	−0.02^*^	0.01^*^	−0.03^*^	0.05^*^	0.11	0.09	0.03^*^	0.01^*^	0.07^*^	0.02^*^	0.00^*^
Intramuscular fat	0.08	0.11	0.12	0.07^*^	0.10	0.17	0.16	0.13	0.12	0.16	0.14	0.12
Muscle depth	−0.09	−0.11	−0.16	−0.10	−0.05^*^	0.10	0.00^*^	−0.02^*^	0.00^*^	−0.01^*^	−0.03^*^	−0.03^*^

^1^MY, methane yield; MIL, methane intensity liveweight; MIC, methane intensity carcass weight; MADG, methane intensity average daily gain; CH_4_:CO_2_, CH_4_:CO_2_ ratio; RMP_adg_, methane adjusted for average daily gain; RMP_cw_, methane adjusted for carcass weight; RMP_lw,_ methane adjusted for liveweight; RMP_e_, methane adjusted for energy intake; RMP_ca_, methane adjusted for carcass weight and average daily gain; RMP_la_, methane adjusted for liveweight and average daily gain; RMP_el_, methane adjusted for energy intake and liveweight.

^2^Scale of 1 (small) to 10 (large).

^3^Scale of 1 to 15. A score of 1 represents poor muscling, and a score of 15 represents excellent muscling.

^4^Scale of 1 to 15. A score of 1 represents poor conformation or a lean carcass, and a score of 15 represents a well conformed or fat carcass.

^5^Hind quarter, development of hind quarter.

^*^ *P* ≥ 0.05.

The partial correlations amongst and between the methane ratio and residual traits, having adjusted for animal sex and breed, are in Table 8. All the residual methane traits were strongly positively correlated with each other (0.91 to 0.99), with the Spearman rank correlations never differing by more than 0.02 from the reported Pearson correlations. Of all the residual methane production traits, RMP_energy_ and RMP_el_ had the strongest correlation with each other (0.99), but also had the weakest correlation with methane production (0.88 to 0.87). Similarly, the ratio traits were all positively correlated with each other (0.59 to 0.86), as well as with the residual methane production traits (0.58 to 0.97). Methane intensity (i.e., ADG as the denominator) had the weakest correlation with the other ratio traits (0.59) and residual methane traits (0.58).

## Discussion

The growing commitment to environmental responsibility, particularly in food production, has intensified the focus on reducing greenhouse gas emissions, including enteric methane production from ruminants. In Ireland, the large cattle population is a notable contributor to the national greenhouse gas emissions inventory, with enteric methane being the second largest contributor to total greenhouse gas emissions (EPA, 2023). Despite the multiple definitions currently used to express methane emissions from ruminants, there is no consensus on the most appropriate definition (de Haas et al., 2017). Seven different definitions of residual methane were explored in the present study, each varying just in the number and combination of the performance traits included in the regression model. By including a performance trait as a covariate in the statistical modeling of methane, the model residuals (i.e., residual methane) are forced to be independent of those traits within the dataset where the model was fitted—this can be particularly useful for quantifying the variability that exists in a population independent of these factors. The objective of the present study was to quantify the extent of this variability but also explore the inter-relationships between a series of traditional and novel methane traits along with the relationship between these traits and performance traits. There is likely to be no single best definition of a methane trait that is best suited to all intended use cases, but at least, knowing the relationships between the different traits may help inform this decision.

### Biological interpretation of regression coefficients in residual traits

The expectation is that animals who consume more, grow faster and/or are heavier should, on average, produce more methane. Corroborating this, Min et al. (2022) and Renand et al. (2019) both reported ADG in beef cattle to be positively associated with methane production. Substantiating this observation, ADG was positively associated with methane production for heifers and steers in the present study and this association remained independent of inter-animal differences in either carcass weight or liveweight. Once, however, energy intake was included in the statistical model of daily methane production, ADG was no longer associated with methane production, suggesting ADG does not provide additional explanatory value for methane production over energy intake but also that the observed association between ADG and methane production is mediated through the association between feed intake and methane production; the correlation between ADG and energy intake in the present study was 0.58. In fact, energy intake was more strongly correlated with methane production (0.48) than was the correlation between ADG and methane production (0.26); the coefficient of variation in energy intake and ADG in the present study was 13.15 and 21.33, respectively.

When liveweight was the only performance trait included in the regression model for methane (i.e., RMP_lw_), the model regression coefficient indicated that each 10 kg increase in liveweight was, on average, associated with just a 3-g increase in methane production per day. This is consistent with reported values from other cattle studies where a 10-kg increase in liveweight was associated with an increase in methane produced per day of 2.3 to 5.0 g (Bird-Gardiner et al., 2017). Meanwhile, the regression model solutions when energy intake was the only performance trait included in the regression model for methane (i.e., RMP_energy_) indicated that each 10 MJ/d increase in energy intake was associated with, on average, an 11.9-g increase in methane produced per day.

The coefficient of determination for daily methane production in the present study (0.31 to 0.43) is similar to that reported by Bird-Gardiner et al. (2017) for feedlot steers (0.19 to 0.45) where their model included both liveweight and DMI. Notably, RMP_el_ in the present study had the highest R^2^ and lowest RMSE of all residual methane traits examined. Nonetheless, the RMSE of this model was still 31.13 g/d which signifies that the difference between the highest 10% on RMP_el_ was, on average, 54.60 g/d higher than those animals that were average on RMP_el_; this equates to 6.5 kg less methane production per animal over a 120-d finishing period for the same feed intake and liveweight.

### Breed effects

The animals recruited to the testing program were progeny of the genetically elite young artificial insemination sires entering the Irish breeding program for each respective breed, so should therefore be a good representation of those breeds. Nonetheless, these sires were elite on the Irish total merit indexes so inference to actual breed effects, especially for other countries, should be undertaken with caution. Multiple studies have documented inter-breed differences in methane production (Rooke et al., 2014; De Mulder et al., 2018; Flay et al., 2019). These studies compared just 2 breed groups, while the present study compared multiple breeds from a larger cohort of animals albeit extracting the breed effects from crossbred data. Consistent with Rooke et al. (2014), Limousins produced less methane per day than Aberdeen Angus cattle, while De Mulder et al. (2018) and Flay et al. (2019) both reported that Holsteins produced more methane than both Belgian Blues and Jerseys. Nonetheless, the ranking of breeds in terms of methane production varied in the present study depending on the definition of RMP although the observed impact was typically rescaling rather than actual breed reranking. Angus cattle produced, on average, 35.89 g more methane daily than Limousins, but only produced 7.68 g/d more than Limousins when energy intake and liveweight were included in the statistical model. Meanwhile, the model regression of methane on Jersey breed proportion increased from 18.21 to 25.06 g/d when energy intake and liveweight were included in the model. This suggests that different traits may explain methane production differently across breeds.

### Correlations among the methane traits

Irrespective of the definition of the residual methane trait, the correlations among all 7 residual traits were always stronger than 0.90 while the correlations between the residual traits and the ratio traits were all stronger than 0.80; the exception was methane intensity defined using ADG in the denominator, which had correlations of 0.58 to 0.72 with the residual methane traits. Correlations of similar strength have been reported in the cattle literature among the residual methane traits (Bird-Gardiner et al., 2017) and between residual methane and ratio traits (Bird-Gardiner et al., 2017; Richardson et al., 2021; Smith et al., 2021). The strong correlations between methane production and the ratio traits were not unexpected because of the part-whole statistical relationship that exists between the ratio traits; methane production was the numerator in all ratio traits, and the coefficient of variation in methane production was 16.4% relative to the other traits (i.e., energy intake, liveweight, carcass weight, and carbon dioxide production) where the coefficient of variation ranged from 11.4% to 12.7%, with the exception of ADG which had a coefficient of variation of 21.3%. The numerator or denominator of ratio traits with the greatest variability usually exerts a stronger influence on the ratio and thus is usually the most correlated with the ratio trait (Adolph and Hardin, 2007).

As the phenotypic correlations among the RMP traits were less than unity, some reranking of animals would be expected depending on which definition was used to classify animals. In the present study, which included 874 animals with RMP phenotypic values, 11 animals that were ranked in the top 20% phenotypically for RMP_energy_ (i.e., 11 out of 175 animals) did not rank in the top 20% when evaluated for RMP_el_. Of the animals that ranked in the top 20% phenotypically for RMP_adg_, 43 animals did not rank in the top 20% (i.e., 43 out of 175) phenotypically for RMP_el_; these all suggest ranking differences between methane efficiency percentiles when based on different RMP traits.

### Correlations with feed behavior traits and subjective skeletal and muscular scores

The positive correlation between energy intake per feed event and methane production (0.11), and negative association between methane production and daily feed events (−0.10) among animals that have the same feed intake and liveweight, indicates that animals who eat less often per day, and consume more energy in each visit, are associated with producing more methane daily. Sepulveda et al. (2022) reported phenotypic and genetic correlations between eating time per day and methane production in Australian ewes, but such correlations have not been previously cited for beef cattle. The number of feed events per day has been shown to have a positive correlation with residual feed intake, indicating that more feed-efficient animals tend to eat more often (Kelly et al., 2020). Greater feed efficiency is associated with lower feed intake overall (Berry and Crowley, 2013), which helps explain why more feed-efficient animals may produce less gross methane (Nkrumah et al., 2006).

The positive association between all the skeletal traits with methane production in the present study supports the hypothesis that larger animals, on average, produce more methane (Herd et al., 2014), consistent also with the positive correlation between liveweight and methane production in the present study. It was expected that RMP_el_ would not be associated with any of the skeletal traits, but it was correlated with both chest width and chest depth. It has been hypothesized that chest width is an indicator of rumen volume in dairy cattle (Williams et al., 2022) while rumen size, as well as the retention time of feed in the rumen, have been shown to be associated with methane yield in sheep (Goopy et al., 2014).

### Correlations with performance traits

Both RMP_energy_ and RMP_el_ were the only methane residual traits not correlated with either energy intake, carcass weight, liveweight or ADG. Meanwhile RMP_adg_ was correlated with energy intake and liveweight. This suggests that variation in energy intake and animal size, even among animals with similar growth rates, was still associated with methane production, while when methane was adjusted for energy intake and liveweight, the associations with the production traits were negligible (Bird-Gardiner et al., 2017); this implies that once the direct effect of energy consumed is accounted for, the residual variation in methane production cannot be further explained by differences in growth efficiency. Given that the animal breed was included as a covariate in the statistical model, the relationship between the traits is independent of breed.

## Conclusion

A total of 13 different traits that included daily methane production were generated of which 5 were based on ratios and 7 were represented as the residuals from a statistical model that accounted for the inter-animal variability in a series of different combinations of performance traits. The ranking of animals for the residual-based traits were all very similar and even the ranking of animals ranked on the residual traits versus the ratio traits was also relatively consistent. Hence, the actual most appropriate definition of methane will result in the context from which it will be used, but at least, it can be done so with the knowledge that the ranking of breeds and animals will be relatively similar irrespective of definition. Whether this conclusion holds for genetic selection needs to be further explored since the present study only evaluated phenotypic association and these may not necessarily translate to genetic correlations.

## Supplementary Material

skae346_suppl_Supplementary_Table_S1

## Abbreviations

ADG: average daily gain
CH_4_: enteric methane
CO_2_: carbon dioxide
DMI: dry matter intake
EUROP: European Union Beef Carcass Classification System
ICBF: Irish Cattle Breeding Federation
RMP: residual methane production
RMSE: root mean square error
TMR: total mixed ration
