# Supplementary material for: Associations between a range of enteric methane emission traits and performance traits in indoor-fed growing cattle
Source: J Anim Sci. 2024 Nov 8;102:skae346. doi: 10.1093/jas/skae346 (PMC11641421; doi:10.1093/jas/skae346)
Supplement: skae346_suppl_Supplementary_Table_S1 [file skae346_suppl_supplementary_table_s1.docx]

**Supplementary Table 1.** Number of animals with recorded measures for each trait.

|  | Steer | Heifer | Bull TMR | Bull Concentrates |
| --- | --- | --- | --- | --- |
| No. Animals | 481 | 320 | 73 | 65 |
| Methane, g/d | 481 | 320 | 73 | 65 |
| Carbon dioxide, g/d | 481 | 320 | 73 | 65 |
| ADG, kg/d | 481 | 320 | 73 | 65 |
| Energy intake, ME/d | 481 | 320 | 73 | 65 |
| Liveweight, kg | 481 | 320 | 73 | 65 |
| Carcass weight, kg | 481 | 320 | 73 | 65 |
| Carcass fat, scale 1-15 | 481 | 320 | 73 | 65 |
| Carcass conformation, scale 1-15 | 481 | 320 | 73 | 65 |
| Fat depth, mm | 290 | 238 | 49 | 65 |
| Muscle depth, mm | 290 | 238 | 49 | 65 |
| Intramuscular fat, % | 290 | 238 | 49 | 65 |
| Methane yield, g CH4/MJ | 481 | 320 | 73 | 65 |
| Methane yield, g CH4/kg DMI | 481 | 320 | 73 | 65 |
| CH_4_:CO_2_ ratio, g CH_4_/g CO_2_ | 481 | 320 | 73 | 65 |
| MIL, g CH_4_/kg lw | 481 | 320 | 73 | 65 |
| MIC, g CH_4_/kg cw | 481 | 320 | 73 | 65 |
| MADG, g CH_4_/kg ADG | 481 | 320 | 73 | 65 |
| Eating time/day, min | 481 | 320 | 73 | 65 |
| Energy eat rate, MJ/min | 481 | 320 | 73 | 65 |
| Daily feed events | 481 | 320 | 73 | 65 |
| MEI per feed event | 481 | 320 | 73 | 65 |
| Width at withers | 243 | 280 | 33 | 42 |
| Width behind withers | 243 | 280 | 33 | 42 |
| Loin development | 243 | 280 | 33 | 42 |
| Development of hind quarters | 243 | 280 | 33 | 42 |
| Thigh width | 243 | 280 | 33 | 42 |
| Chest depth | 243 | 280 | 33 | 42 |
| Chest width | 243 | 280 | 33 | 42 |
| Height of withers | 243 | 280 | 33 | 42 |
| Length of back | 243 | 280 | 33 | 42 |
| Pelvic length | 243 | 280 | 33 | 42 |
| Width of hips | 243 | 280 | 33 | 42 |
| RMP_lw,_ g/d | 481 | 320 | 73 | - |
| RMP_cw_, g/d | 481 | 320 | 73 | - |
| RMP_adg_, g/d | 481 | 320 | 73 | - |
| RMP_energy_, g/d | 481 | 320 | 73 | - |
| RMP_el_, g/d | 481 | 320 | 73 | - |
| RMP_la_, g/d | 481 | 320 | 73 | - |
| RMP_ca_, g/d | 481 | 320 | 73 | - |

^1^ ADG = Average daily gain, MIL = methane intensity liveweight, MIC = methane intensity carcass weight, MADG = methane intensity average daily gain, MEI = metabolisable energy intake, RMP_lw_  = methane adjusted for liveweight, RMP_cw_ = methane adjusted for carcass weight, RMP_adg_ = methane adjusted for average daily gain, RMP_energy_ = methane adjusted for energy intake, RMP_el_ = methane adjusted for energy intake and liveweight, RMP_el_ = methane adjusted for energy intake and liveweight, RMP_la_ = methane adjusted for liveweight and average daily gain, RMP_ca_ = methane adjusted for carcass weight and average daily gain. Residual traits were not calculated for bulls on a concentrate diet. * = energy eat rate for bulls on a concentrate diet was for the concentrates from the Insentec feeder only.
